# Tethered Balloon Cluster Deployments and Optimization for Emergency Communication Networks

**DOI:** 10.3390/e26121071

**Published:** 2024-12-09

**Authors:** Mingyu Guan, Zhongxiao Feng, Shengming Jiang, Weiming Zhou

**Affiliations:** College of Information Engineering, Shanghai Maritime University, Shanghai 201306, China; 202330310132@stu.shmtu.edu.cn (M.G.); fzxfire@163.com (Z.F.); xiaosheng1418@163.com (W.Z.)

**Keywords:** emergency communication, multiple tethered balloons, coverage rate, energy efficiency, multi-objective particle swarm optimization (MOPSO), network performance

## Abstract

Natural disasters can severely disrupt conventional communication systems, hampering relief efforts. High-altitude tethered balloon base stations (HATBBSs) are a promising solution to communication disruptions, providing wide communication coverage in disaster-stricken areas. However, a single HATBBS is insufficient for large disaster zones, and limited resources may restrict the number and energy capacity of available base stations. To address these challenges, this study proposes a cluster deployment of tethered balloons to form flying ad hoc networks (FANETs) as a backbone for post-disaster communications. A meta-heuristic-based multi-objective particle swarm optimization (MOPSO) algorithm is employed to optimize the placement of balloons and power control to maximize target coverage and system energy efficiency. Comparative analysis with a stochastic algorithm (SA) demonstrates that MOPSO converges faster, with significant advantages in determining optimal balloon placement. The simulation results show that MOPSO effectively improves network throughput while reducing average delay and packet loss rate.

## 1. Introduction

When extreme natural disasters occur, damage to power systems and network infrastructure often leads to severe communication disruptions, which endangers health security, life safety, and social stability [1]. Emergency communication networks are critical in the event of disasters, as the speed of rescue operations and the efficiency of recovery efforts heavily depend on the availability of on-site communications [2]. Traditional emergency communication methods, such as vehicle-mounted base stations and unmanned aerial vehicles (UAVs), cannot fully meet the needs of certain rescue scenarios. Satellite services offer limited bandwidth for large data transfers and are vulnerable to attack during military conflicts [3]. Given the urgency of disaster response, high-altitude platform stations (HAPSs), with their rapid deployment capabilities and lack of dependence on large-scale infrastructure, combine the strengths of terrestrial and satellite systems and play a critical role in disaster mitigation and post-disaster relief [1,4].

High-altitude tethered balloon base stations (HATBBSs), with excellent airborne capability and reliable service, provide long-term, continuous communication coverage over wide areas. They can be rapidly deployed to target areas through airborne vehicles, such as planes and helicopters, providing efficient connectivity for environments lacking infrastructure and constructing space-air-ground-sea integrated networks (SAGSINs) [5]. Therefore, optimizing the deployment of HATBBSs to give full play to their performance is an issue of concern. In open areas with fewer geographic obstacles, HATBBSs leverage their altitude to provide extensive wireless communication coverage. Suspended at heights of hundreds of meters, they can avoid interference from terrain and obstacles, extending wireless signals to remote and hard-to-reach areas, thereby enabling reliable communications.

Wireless communication in complex environments, particularly in distant seas [6] and rural regions, presents significant challenges. Rough terrain and varied atmospheric conditions can lead to multi-path effects, causing signal attenuation, interference, and loss. Optimizing the deployment heights of HATBBSs can effectively reduce these multi-path effects, ensuring more direct and stable signal propagation. Additionally, environmental characteristics, such as humidity, atmospheric absorption, and marine salt spray, will attenuate radio waves, necessitating adjustments to transmit power and frequency band selection to reduce co-channel interference [7,8] and maximize network coverage and energy efficiency.

This paper offers an in-depth investigation and optimization of the deployment problem of balloons to ensure sufficient communication. The main contributions of our research are summarized as follows:

(1) The complex dynamic characteristics of balloons in the air are considered to reflect practical application scenarios more comprehensively, especially the mobility characteristics. To capture and predict the positional changes of balloons accurately, we utilize a Gauss–Markov mobility model, which is well-suited for representing the continuous but partially random movement patterns of high-altitude platforms.

(2) Based on the constructed system model and the corresponding constraints on distance, coverage, frequency band, and power, the balloon deployment problem is transformed into a multi-objective optimization problem to maximize target coverage and system energy efficiency.

(3) Multi-objective particle swarm optimization (MOPSO) is applied to address the resulting mixed-integer nonlinear programming problem. Given the higher computational complexity of MOPSO, a low-complexity stochastic algorithm (SA) is also introduced as a benchmark to provide a comprehensive comparative performance analysis.

The rest of this article is organized as follows. Section 2 briefly reviews related research. Section 3 introduces the system model and formulates the balloon deployment problem. Section 4 details the methodology used to solve the multi-objective optimization problem. Section 5 presents simulation validations and an analysis of the results. Finally, the paper is concluded in Section 6.

## 2. Related Works

Ref. [1] analyzes network performance regarding delay, throughput, and signal-to-noise ratio (SNR), showing that tethered balloons can efficiently enhance emergency communication with minimal delays and strong throughput. The advantages include rapid deployment, low cost, and reliable service; however, challenges include coverage limitations and susceptibility to specific disaster types. In [4], the authors argue that aerial platforms represented by tethered balloons play an integral role in rescue in major disaster scenarios and simulate the performance of communication services. The authors of [9] state that tethered balloons are suitable for disasters except for storms and calculate their coverage and path loss at different distances. In [10], a multi-antenna tethered balloon was used as a repeater between a UAV high-altitude platform and a ground base station for an interference alignment technique. It is shown that there is an optimal balloon altitude that maximizes the overall rate of balloon-ground communication. A joint layered deployment of tethered balloons and UAVs is proposed in [11]. The optimal and greedy algorithms are employed to minimize the number of tethered balloons and UAVs deployed while providing effective coverage. Ref. [12] explores the relationship between coverage, propagation loss, and height using the Hata propagation model. A distributed deep Q-learning network optimization scheme based on resource allocation and joint UAV and tethered balloon placement control is discussed in [13] to maximize the total rate of this network. In [14], a UAV and tethered balloon-assisted allocation strategy is utilized to improve end-to-end throughput of the terrestrial infrastructure network. Ref. [15] proposed to take advantage of the low cost and flexible deployment of tethered balloons to deploy them in mountainous areas with extremely complex terrain and simulated their coverage quality and propagation loss using the Longley–Rice model. Ref. [16] uses a stochastic geometric approach and a user association strategy to optimize the location of tethered unmanned aerial vehicles (T-UAVs) to achieve cellular network coverage in areas where users congregate, concluding that T-UAVs trade-off mobility for an increase in coverage probability. Table 1 summarizes the contributions between this paper and the extant studies.

## 3. System Model and Problem Formulation

### 3.1. Geometrical Model

In this section, a high-altitude tethered balloon communication system model is constructed in which balloons are used as temporary airborne base stations to provide communication services to end-users. In this system, the air-to-air communication link between balloon base stations uses LTE backhaul link technology to achieve self-organized networking [17]. The communication between balloon base stations and ground users or ground base stations adopts the air-to-ground access link, using LTE technology, to provide wireless access service for ground equipment. A multi-hop, multi-link network architecture can be deployed between two distant ground stations. The network is formed by deploying multiple balloon base stations in the intermediate area, with each balloon base station acting as a relay node, supporting multi-path transmission to improve reliability and using the AODV routing protocol [18,19] to achieve long-distance multi-hop communication.

**Figure 1:**
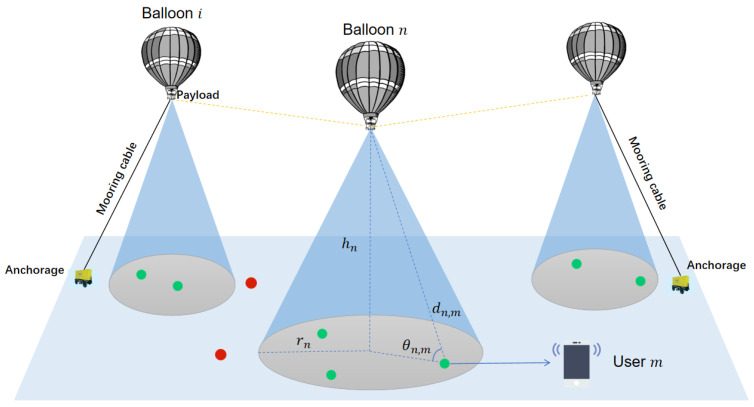
Wireless emergency communication system supported by HATBBSs.

As shown in Figure 1, there are *n* balloons in this system providing data transmission to *m* users. The users are randomly distributed in a two-dimensional plane $D=\left\{\left(x,y\right)|x_{min}\leq x\leq x_{max},y_{min}\leq y\leq y_{max}\right\}$, while balloons are deployed in a 3D area $P=\left\{\left(x,y,h\right)|x_{min}\leq x\leq x_{max},y_{min}\leq y\leq y_{max},h_{min}\leq h\leq h_{max}\right\}$. Let $N=\left\{\begin{array}{c} 1,2,\cdots ,N \end{array}\right\}$ represent the set of balloon base stations. Let $M=\left\{\begin{array}{c} 1,2,\cdots ,M \end{array}\right\}$ represent the set of users; the set of users covered by balloon *n* can be expressed as $M_{n}=\left\{\begin{array}{c} M_{1},M_{2},\cdots ,M_{n} \end{array}\right\}$. In addition, to decrease co-channel interference between neighboring balloon base stations, let $B=\left\{\begin{array}{c} 1,2,\cdots B \end{array}\right\}$ denote the set of available frequency bands. Each base station can only use at most one band to provide communication for multiple users. The frequency band of base station *n* is denoted by $b_{n}$. The set of base stations using band *b* is $N_{b}$ [20].

The 3D coordinates of the nth balloon base station and the mth user can be expressed, respectively, as $P_{n}=\left\{\begin{array}{c} x_{n},y_{n},h_{n}|x_{min}\leq x_{n}\leq x_{max},y_{min}\leq y_{n}\leq y_{max},h_{min}\leq h_{n}\leq h_{max} \end{array}\right\}$ and $D_{m}=\left\{\begin{array}{c} x_{m},y_{m}|x_{min}\leq x_{m}\leq x_{max},y_{min}\leq y_{m}\leq y_{max} \end{array}\right\}$. Therefore, the distance between balloon *n* and user *m* is $d_{m,x}$ can be given by

(1) $$d_{n,m}=\sqrt{{\left(x_{n}-x_{m}\right)}^{2}+{\left(y_{n}-y_{m}\right)}^{2}+{h_{n}}^{2}}.$$

### 3.2. Mobility Model

Flying ad hoc networks (FANETs) consisting of HATBBSs exhibit some mobility in the troposphere due to the wind field and turbulence. This mobility leads to changes in the speed and position of base stations, which may degrade the network performance. The movement of balloons is both somewhat predictable and subject to unexpected factors. In order to accurately capture the complex dynamics of airborne mobile nodes and to facilitate efficient communication relaying, the Gauss–Markov Mobility Model (GMM) has been proposed and widely used [21].

In this model, a node is initially assigned a specific speed, direction, and pitch angle. Subsequently, the node’s velocity, direction, and pitch angle at the next moment depend on the state at the current moment and are updated after a fixed time interval [22]. This approach balances complete randomness and complete determinism, where the parameter α determines the degree of randomness of the node’s movement. When α = 0, the model exhibits no memory properties; when α = 1, the model exhibits full memory properties. Specifically, the model uses the following three equations to calculate the velocity v(t), direction o(t), and pitch angle p(t) at moment *t*, respectively, which are based on the corresponding values at moment (t−1),

(2) $$v\left(t\right)=\alpha v\left(t-1\right)+\left(1-\alpha \right)\bar{v}+\sqrt{1-\alpha^{2}}v_{x_{t-1}},$$

(3) $$o\left(t\right)=\alpha o\left(t-1\right)+\left(1-\alpha \right)\bar{o}+\sqrt{1-\alpha^{2}}o_{x_{t-1}},$$

(4) $$p\left(t\right)=\alpha p\left(t-1\right)+\left(1-\alpha \right)\bar{p}+\sqrt{1-\alpha^{2}}p_{x_{t-1}},$$

where $\bar{v}$, $\bar{o}$, and $\bar{p}$ are mean constants for velocity, direction, and pitch angle, respectively, and $v_{x_{t-1}}$, $o_{x_{t-1}}$, and $p_{x_{t-1}}$ are random variables obeying Gaussian distribution. The node’s position at the moment *t* is jointly determined by the position, velocity, direction, and pitch angle at the moment (t−1) [21,22]. ∀n∈N, the 3D coordinates of node *n* are updated as follows:

(5) x(t)=x(t−1)+v(t−1)cos(o(t−1))cos(p(t−1)),

(6) y(t)=y(t−1)+v(t−1)sin(o(t−1))cos(p(t−1)),

(7) h(t)=h(t−1)+v(t−1)sin(p(t−1)).

### 3.3. Channel Model

#### 3.3.1. Air-to-Air Channel Model

The balloon-to-balloon channel is virtually unobstructed and dominated by line-of-sight (LoS) links, so the propagation loss between balloon *n* and balloon *i* lends itself to free-space propagation loss (FSPL),

(8) $$L_{LoS}^{n,i}={\left(\frac{4\pi d_{n,i}f_{c}}{c}\right)}^{2},$$

where $d_{n,i}$ represents the distance between neighboring balloons *n* and balloon *i* and $d_{n,i}\geq d_{min}$. $d_{min}$ is the minimum safe distance to ensure that balloons do not collide. $f_{c}$ denotes the carrier frequency, and *c* is the speed of light.

#### 3.3.2. Air-to-Ground Channel Model

The channel between balloons and users has not only LoS links but also non-line-of-sight (NLoS) links. The propagation of wireless signals in complex environments is affected by several factors. Firstly, buildings or other reflective surfaces such as the sea have high reflectivity, which tends to cause multi-path effects in signal propagation. Secondly, atmospheric humidity, shadowing effects, and other environmental factors may seriously attenuate the strength and quality of signals. Therefore, NLoS path loss is usually much larger in complex environments than LoS path loss.

Considering the above analyses, the air-to-ground (ATG) propagation channel model is a weighted propagation loss model that combines LoS probability and NLoS probability. This model is particularly suitable for calculating the path loss of the communication system between aerial platforms and users. The probabilities of LoS and NLoS links between balloon *n* and user *m* can be expressed as, respectively,

(9) $$P\left(LoS,\theta_{n,m}\right)=\frac{1}{1+a\;exp(-b\left(\theta_{n,m}-a\right))},$$

(10) $$P\left(NLoS,\theta_{n,m}\right)=1-P\left(LoS,\theta_{n,m}\right),$$

where $\theta_{n,m}$ represents the elevation angle at which base station *n* establishes a LoS link with user *m*. $P(LoS,\theta_{n,m})$ is the probability that a direct LoS propagation path exists between the transmitter and receiver. Its expression depends mainly on the following factors: elevation angle dependence and environmental factors. When the flight altitude is higher and the elevation angle is higher, $P(LoS,\theta_{n,m})$ is usually higher. $P(LoS,\theta_{n,m})$ is usually lower when environmental factors such as urban density, building height, and vegetation cover play a stronger role. *a* and *b* are constant values that depend on the type of environment, e.g., urban, rural, suburban, etc. *a* reflects the density of buildings. A larger value of *a* indicates a higher density of buildings. *b* is related to the geometrical distribution of the buildings. Larger values of *b*, $P(LoS,\theta_{n,m})$ vary more rapidly with elevation. The path loss of the LoS link and the NLoS link between balloon *n* and user *m* can be expressed as, respectively,

(11) $$L_{LoS}^{n,m}={\left(\frac{4\pi d_{n,m}f_{c}}{c}\right)}^{2}\;\eta_{LoS},$$

(12) $$L_{NLoS}^{n,m}={\left(\frac{4\pi d_{n,m}f_{c}}{c}\right)}^{2}\;\eta_{NLoS}.$$

FSPL usually assumes that signals propagate in a vacuum without any obstacles or interference. However, in real-world environments, signals are affected by various factors such as atmospheric absorption, scattering, diffraction, reflection, etc., which can lead to additional losses. $\eta_{LoS}$ and $\eta_{NLoS}$ are the additional loss terms for LoS and NLoS links propagating with respect to FSPL due to atmospheric absorption and obstacle occlusion, respectively [23]. The weighted average path loss between balloon *n* and user *m* is given by

(13) $$L_{n,m}=P\left(LoS,\theta_{n,m}\right)\;L_{LoS}^{n,m}+P\left(NLoS,\theta_{n,m}\right)\;L_{NLoS}^{n,m}.$$

The Signal-to-Interference-plus-Noise Ratio (SINR) accurately reflects the reality of the presence of interference in a wireless communication network. The success of a communication link does not depend on the presence of other transmitting links but rather on whether the total interference generated by all the links in the area transmitting data is greater than a predetermined threshold. Define $\gamma_{n,m}$ to denote the SINR of the receiving node *m* when the balloon *n* sends data to the user *m*, and *m* is considered to be covered when $\gamma_{n,m}$ exceeds a threshold denoted by Λ.

(14) $$\gamma_{n,m}=\frac{P_{n,m}\cdot g_{n,m}}{\sum_{i\neq n}P_{i,m}\cdot g_{i,m}+\eta B_{n}},$$

(15) $$C_{n,m}=\left\{\begin{array}{cc} 1, & \mathrm{if}\;\;d_{n,m}\leq \sqrt{h_{n}^{2}+r_{n}^{2}}\;\;\mathrm{and}\;\;\gamma_{n,m}\geq \Lambda ,\\ 0, & \mathrm{otherwise}, \end{array}\right.$$

where $P_{n,m}$ and $g_{n,m}$ are the signal transmit power and channel gain between base station *n* and user *m*, respectively. η is the power spectral density of Gaussian white noise [24]. $B_{n}$ is the bandwidth used by base station *n*. $r_{n}$ and $h_{n}$ represent the maximum coverage radius and maximum vertical height of base station *n*. $C_{n,m}$ represents the coverage of user *m* by balloon *n*.

Therefore, by Shannon’s theorems, the maximum reception rate of user *m* can be calculated as

(16) $$R_{m}=B_{n}log_{2}\left(1+\gamma_{n,m}\right).$$

### 3.4. Objective Function

The objectives of the deployment are to maximize the target coverage $f_{1}$, defined as the ratio of the number of covered users to the total number of users, and the system energy efficiency $f_{2}$, defined as the sum of the reception rates of all covered users divided by the sum of the transmit power of all balloons. Thus, the fitness function for the balloon deployment problem can be formulated as Equation (18). X represents an assignable deployment scenario that satisfies the constraints (C1–C12).

(17) $$\left\{\begin{array}{cc} f_{1}= & \frac{\sum_{m\in M}\sum_{n\in N}C_{n,m}}{M},\\ f_{2}= & \frac{\sum_{m=1}^{M}R_{m}}{\sum_{n=1}^{N}P_{n}}, \end{array}\right.$$

(18) $$F\left(X\right)=\max_{\left\{P_{n}\right\},N,M,\left\{P_{n}\right\}}\left[f_{1}\left(X\right),f_{2}\left(X\right)\right]$$

(18a)s.t.C1:Pn∈P,∀n∈N,(18b)C2:Dm∈D,∀m∈M,(18c)C3:hmin≤hn≤hmax,(18d)C4:dn,i≥dmin,∀n,i∈N,n≠i,(18e)C5:Pmin≤Pn≤Pmax,(18f)C6:∑n∈NMn≤M,(18g)C7:Mn∩Mi=⌀,∀n,i∈N,n≠i,(18h)C8:⋃b∈BNb=N,(18i)C9:Nb⋂Ne=⌀,∀b,e∈B,b≠e,(18j)C10:∑n∈NBn≤B,(18k)C11:Cn,m∈0,1,∀n∈N,m∈M,(18l)C12:γn,m≥Cn,mΛ,∀n∈N,m∈M.

In Equation (18), it can be seen that as the balloon deployment height increases, the coverage increases, while the LoS probability and path loss increase and the user reception rate decreases accordingly when the balloon transmit power is determined, which can lead to a reduction in energy efficiency, and thus the two objective functions are in conflict with each other. The problem belongs to mixed integer nonlinear programming; improving one of them must lead to the degradation of the other. It is an NP-hard problem, which is difficult to solve by using traditional methods. Therefore, it is considered to be solved by using MOPSO in Section 4.

## 4. Methodology

The multi-objective particle swarm optimization (MOPSO) algorithm, inspired by the flocking behavior of birds, is designed to optimize two or more conflicting objectives simultaneously, which means that an increase in the performance of one objective function will result in a decrease in the performance of the remaining objective functions. While traditional Particle Swarm Optimization (PSO) algorithms are designed to solve a single objective function, MOPSO extends PSO to deal with multi-objective optimization problems. Since multi-objective problems usually do not have a unique optimal solution, the method aims to find a set of solutions that consider all the objectives and provide good trade-offs, i.e., the Pareto optimal set (POS). The fitness evaluation of all solutions is based on the multi-objective function of the problem to be optimized. The fitness function is used to measure the performance of the particles under the current deployment scenario.

To measure the superiority of the multi-dimensional solutions, MOPSO introduces a selection mechanism to evaluate the fitness based on Pareto optimality, i.e., Pareto dominance. This dominance provides an objective criterion for comparing the advantages and disadvantages of different solutions, enabling the algorithm to make effective trade-offs between multiple objectives. Specifically, solution $X_{1}$ is said to dominate solution $X_{2}$ when and only when solution $X_{1}$ performs non-inferiorly to $X_{2}$ on all objective functions and strictly outperforms $X_{2}$ on at least one objective function. The relationship between multi-dimensional fitness and Pareto dominance for $X_{1}$ and $X_{2}$ is shown below:

(19) $$\forall \;X_{1},X_{2}\in X,\;X_{1}\;\;dominates\;\;X_{2}\Leftrightarrow \left\{\begin{array}{c} \forall \;\;i\in \left(1,2\right),\;f_{i}\left(X_{1}\right)\geq f_{i}\left(X_{2}\right),\\ \exists \;\;j\in \left(1,2\right),\;f_{j}\left(X_{1}\right)>f_{j}\left(X_{2}\right). \end{array}\right.$$

Assuming that there are *S* particles in search space *X*, MOPSO first initializes and randomly assigns positions to all the particles, which represent potential solutions to the multi-dimensional optimization problem, which are dispersed in the search space expected to maximize the multi-objective function. Each particle possesses four attributes for exploring the search space of the problem: the current velocity of the particle, its current position, the best position experienced by the individual (the optimal solution found by the individual during the iteration process, denoted as $P_{Best}$), and the best position experienced by the group (the best-fitness position among all particles, denoted as $G_{Best}$). Set the particle’s current position to $P_{Best}$. The particle that performs best in both objective functions is selected as the initial $G_{Best}$.

During each iteration, MOPSO updates the velocities and positions of all particles based on the guidance of $P_{Best}$ and $G_{Best}$ (see Equations (20) and (21)) and calculates the fitness to evaluate their current positions to maximize the probability of moving to more optimal solution space and thus obtaining a better fitness. Judge the superiority of the current position $X_{i}$ and $P_{Best[i]}$ based on Pareto dominance: if $X_{i}$ dominates $P_{Best[i]}$ (i.e., $X_{i}$ performs better on at least one objective and is not worse than $P_{Best[i]}$ on all other objectives), update $P_{Best[i]}$ to $X_{i}$; if $P_{Best[i]}$ dominates $X_{i}$, keep $P_{Best[i]}$ unchanged. In the same way, the dominant relationship between $P_{Best}$ and $G_{Best}$ is judged and $G_{Best}$ is updated (see Equations (23) and (24)). This process continues until the maximum number of iterations is reached or enough $G_{Best}$ have been collected to obtain a set of optimal deployment locations finally [25]. The steps of MOPSO can be expressed as Algorithm 1.
**Algorithm 1:** MOPSO for Balloon Deployment Optimization
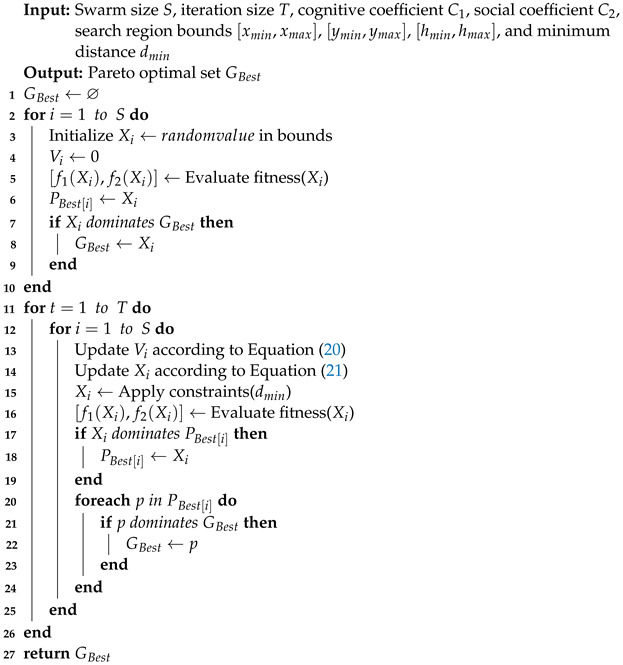



The velocity, position, and individual optimal position of particle *i* in the next iteration are updated by

(20) $$V_{i}\left(t+1\right)={wV}_{i}\left(t\right)+C_{1}R_{1}\left(P_{Best[i]}\left(t\right)-X_{i}\left(t\right)\right)+C_{2}R_{2}\left(G_{Best}\left(t\right)-X_{i}\left(t\right)\right),$$

(21) $$X_{i}\left(t+1\right)=X_{i}\left(t\right)+V_{i}\left(t+1\right),$$

(22) $$w=0.9-0.5\times \frac{t}{T},$$

(23) $$P_{Best[i]}\left(t+1\right)=\left\{\begin{array}{cc} X_{i}\left(t+1\right), & \mathrm{if}\;\;\;X_{i}\left(t+1\right)\;\;dominates\;\;P_{Best[i]}\left(t\right),\\ P_{Best[i]}\left(t\right), & \mathrm{otherwise}, \end{array}\right.$$

(24) $$G_{Best}\left(t+1\right)=\left\{\begin{array}{cc} P_{Best[i]}\left(t+1\right), & \mathrm{if}\;\;\;P_{Best[i]}\left(t+1\right)\;\;dominates\;\;G_{Best}\left(t\right),\\ G_{Best}\left(t\right), & \mathrm{otherwise}. \end{array}\right.$$

In Equations (20) and (21), $V_{i}\left(t\right)$ is the velocity of the ith particle at the tth iteration, $X_{i}\left(t\right)$ is the position of the ith particle at the tth iteration, and $X_{i}\left(t\right)\in R^{3}$. $C_{1}$ and $C_{2}$ are constants defining the cognitive learning coefficient and social learning coefficient of the particle, respectively. $R_{1}$ and $R_{2}$ are random numbers between 0 and 1. $P_{Best[i]}$ is the best position found by particle *i*. *t* and *T* represent the number of current iterations and the total number of iterations, respectively [26]. The inertia weight *w* determines how much momentum the particle velocity maintains during the iteration. Larger inertia weights give the particle a faster velocity and a greater likelihood of performing a global search throughout the solution space, which helps to explore more potential solutions. Smaller inertia weights, on the other hand, give the particles lower speeds and more focus on local search in the current region, which helps to finely optimize the solution. Equation (22) describes a linearly decreasing inertia weighting strategy, which gives the algorithm a stronger global exploration capability initially by gradually decreasing *w* during the iteration process. At the later stage, it will gradually decrease *w* to enhance the local search capability, thus achieving full exploration and fine exploitation of the solution space and improving the solution quality.

We also introduce the stochastic algorithm (SA) as a benchmark comparison for MOPSO. SA is a purely stochastic search optimization method; the core idea is to randomly sample a given search space and find an approximate optimal solution through many iterations and random attempts. Each iteration is independent of the other and generates new random locations, and for each newly generated deployment scenario, two objective function values are computed. If the new solution outperforms the historical optimal solution on both metrics simultaneously, the corresponding optimal record is updated and retained. The main steps of SA are denoted as Algorithm 2.
**Algorithm 2:** SA for balloons deployment locations
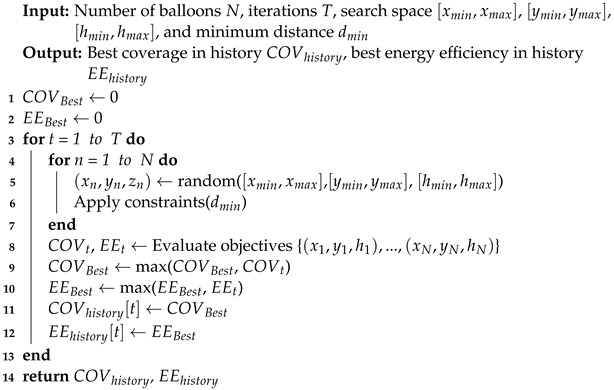



## 5. Simulation Validations and Discussion

### 5.1. Simulation Setup

This experiment uses Python and NS-3 (Network Simulator-3) to verify the performance of the proposed algorithm and system model, respectively. All balloon base stations and users are initially randomly distributed, and all data are taken as the final value of the algorithm after the completion of the current iteration. The values of primary parameters are listed in Table 2.

### 5.2. Simulation Results

**Figure 2:**
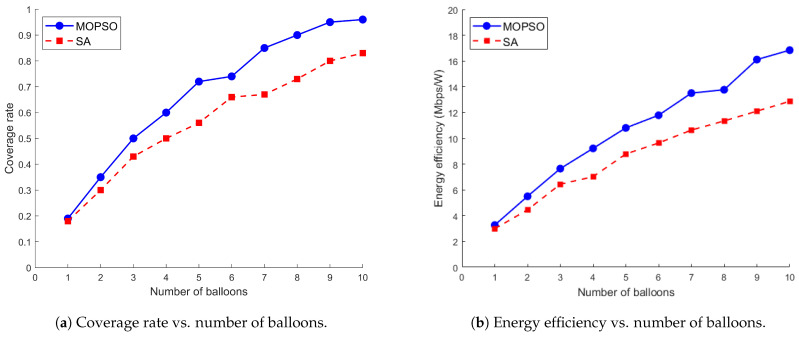
Comparison of MOPSO and SA for coverage rate and energy efficiency.

Figure 2 shows the effect of MOPSO and SA on target coverage rate and system energy efficiency when the number of deployed balloons increases from 1 to 10, and both algorithms show an increasing trend. However, MOPSO consistently shows a significant advantage, with a 19.43% and 25.7% improvement in coverage and energy efficiency, respectively, compared to SA. This considerable performance difference can be explained by the properties of MOPSO and SA. MOPSO can optimize multiple conflicting objective functions simultaneously, maintaining the diversity of solutions during the search process and thus exploring the solution space more efficiently. It can quickly converge to the global optimal solution set through the information exchange between particles and the guidance of the global optimal solution. In contrast, although SA is simple, its completely stochastic nature makes it difficult to effectively utilize the structural information of the problem and often requires a large number of iterations to find a better solution in complex multi-objective optimization.

**Figure 3:**
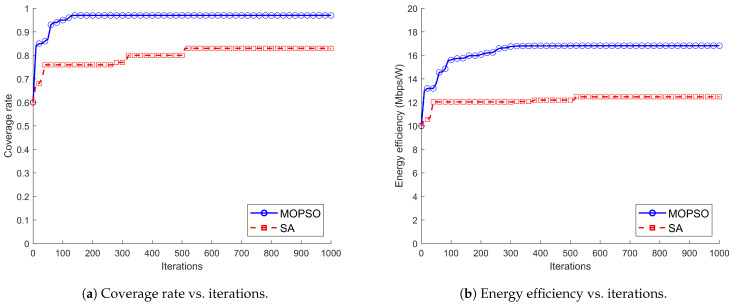
The convergence curves for MOPSO and SA.

Figure 3 illustrates the coverage and energy efficiency versus iterations of MOPSO and SA with a fixed number of balloons of 10. The two algorithms are significantly different in terms of convergence speeds. MOPSO shows a stepwise increase, which means that it finds better solutions at each iteration step and gradually approaches the optimal solution set. After 130 and 300 iterations, the coverage and energy efficiency stabilized at 96% and 16.84 Mbps/W, respectively, which indicates that they found a deployment scheme that maximized the coverage and energy efficiency while satisfying the constraints. The coverage and energy efficiency of SA, although reaching higher values early at about 50 iterations, do not converge and continue to increase in the subsequent 300 and 500 iterations with a slightly upward trend and stabilize at 83% and 12.48 Mbps/W, respectively, after 500 iterations. Because SA generates a new solution randomly at each iteration and cannot use the previous information to guide the search direction, the convergence speed is very slow. There is a large gap between its overall performance and that of MOPSO, which shows a faster convergence speed due to its ability to use inter-particle collaboration and information sharing to search the solution space.

**Figure 4:**
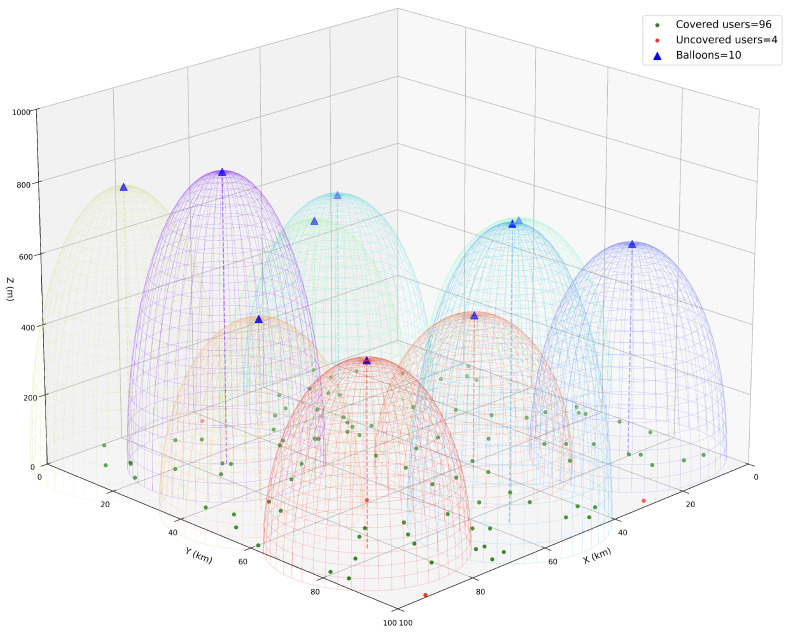
3D visualization of balloons coverage.

Figure 4 depicts the final positions of ten balloons deployed and their coverage under MOPSO calculation after 1000 iterations. It can be seen that the target coverage rate at this point is 96%, which matches the data at the end of MOPSO curves in Figure 2a and Figure 3a. The high-altitude tethered balloon communication system involves several interacting objectives such as energy efficiency and coverage, and MOPSO can effectively balance these objectives to find a more optimal deployment plan. This result is of great significance for the deployment of practical systems, implying that higher system performance can be achieved with MOPSO under the same resource input.

**Figure 5:**
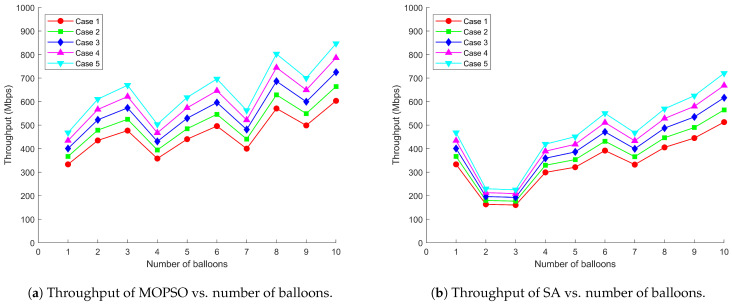
Comparison of MOPSO and SA on throughput under different user distribution scenarios.

**Figure 6:**
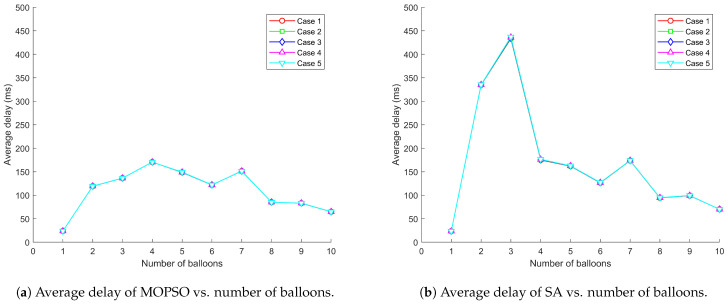
Comparison of MOPSO and SA on average delay under different user distribution scenarios.

**Figure 7:**
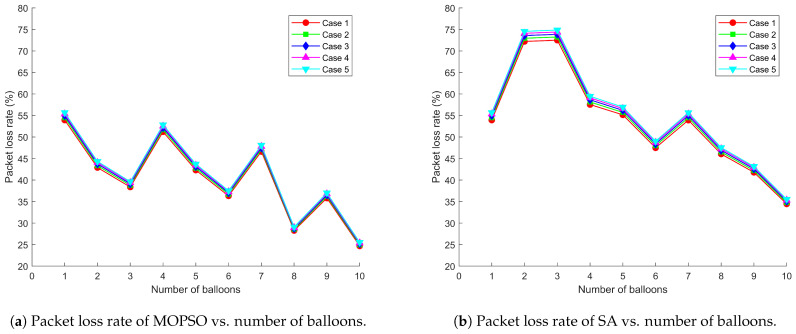
Comparison of MOPSO and SA on packet loss rate under different user distribution scenarios.

Figure 5, Figure 6 and Figure 7 demonstrate the relationship between total throughput, average delay, packet loss rate, and number of balloons when the communication system adopts MOPSO and SA, respectively, under five distribution scenarios for users. The experimental results show that MOPSO achieves a 37.09% improvement in total throughput, a 34.86% decrease in average delay, and a 13.69% reduction in packet loss rate compared to SA. From a physical point of view, these improvements can be attributed to better deployment locations causing better SINR management and more efficient utilization of radio resources. MOPSO always outperforms SA, especially when the number of balloons is high. The performance advantage of MOPSO is mainly attributed to its ability to optimize the deployment locations of balloons. Through a more reasonable deployment scheme, MOPSO can effectively avoid the overlapping of balloon coverage and mutual interference, shorten the data transmission path, and assign users to base stations with higher signal strength through adaptive power control. This optimization directly impacts the physical layer performance by reducing path loss, reducing co-channel interference and improving channel conditions. In contrast, SA may lead to coverage overlap or ground nodes connecting to distant base stations due to the randomness of balloon deployment locations, thus reducing the overall network performance.

It can be observed from Figure 5a–Figure 7a that MOPSO can effectively optimize balloon locations and power allocation to increase the network capacity while keeping the latency and packet loss rate low. This is achieved through optimal spatial diversity exploitation and the efficient management of the radio frequency spectrum. MOPSO tries to find a better combination of locations in each iteration to maximize the coverage and the signal quality and thus improve throughput. However, when the numbers of balloons are 4, 7, and 9, there is a significant drop in throughput. That is attributed to the fact that it may fall into a local optimal solution, resulting in little difference in the location optimization effect for the increased numbers of balloons. From an electromagnetic wave propagation perspective, as the number of balloons increases, the interference in the network increases accordingly, which partially cancels out the performance improvement brought by MOPSO, resulting in a fluctuating increase in throughput. This phenomenon can be explained by the increased probability of co-channel interference and multipath fading effects in denser networks. The adaptive power control mechanism plays a moderating role in this process by dynamically adjusting the transmit power in an attempt to find a balance between coverage and interference control. This power adaptation directly affects the received signal strength and interference levels at user terminals, creating a complex electromagnetic environment that requires careful management. Network performance fluctuations can be regarded as a dynamic outcome reached by these multi-party games. In contrast, as shown in Figure 5b–Figure 7b, the overall performance of SA is not as good as that of MOPSO, which is mainly due to the lack of reasonable planning of balloon locations, resulting in overlapping coverage and causing signal interference and resource wastage. This leads to destructive interference patterns and increased inter-cell interference, degrading the physical layer performance. As the number of balloons increases, this overlap and interference problem becomes more serious, which in turn leads to poor overall network performance.

### 5.3. Discussion

In summary, this study reveals the performance characteristics of MOPSO-based high-altitude balloon networks in terms of coverage, energy efficiency, throughput, delay, and packet loss rate. The physical layer analysis demonstrates how MOPSO effectively manages the complex electromagnetic environment through optimal spatial deployment and power control. The results show that MOPSO, with a faster convergence speed, exhibits significant advantages in optimizing balloon deployment locations and power allocation and can effectively improve performance. This is particularly evident in the improved SINR distribution and reduced electromagnetic interference patterns across the network. The observed non-linear growth and fluctuations in performance reflect the complex interaction between algorithmic optimization, network interference, and adaptive power control. This dynamic equilibrium highlights the multi-objective nature of the high-altitude balloon network optimization problem and the effectiveness of MOPSO in dealing with such complex problems, providing a viable solution for building high-performance and reliable high-altitude communication systems. SA lacks a systematic search strategy and directional guidance, leading to slow convergence and lower-quality solutions, especially when dealing with high-dimensional or multi-objective optimization problems. Future research directions could focus on improving MOPSO to better handle local optimum problems, developing more efficient interference management strategies and exploring the possibility of integrating machine learning techniques into the optimization process. These improvements are expected to further enhance the performance of high-altitude balloon networks and lay the foundation for large-scale deployments.

## 6. Conclusions

This paper investigates the deployment and optimization of HATBBSs for emergency communications. Compared to existing research, this study especially considers the impact of the complex mobility of base stations on network performance. Based on the proposed system model and constraints, the deployment problem is transformed into a multi-objective optimization problem for maximizing coverage and energy conversion, and MOPSO is used to address the problem. By comparing and analyzing with SA, the simulation results confirm that the proposed method shows significant advantages in several key performance indicators: user coverage, system energy efficiency, and total throughput are effectively improved, while average delay and packet loss rate are reduced. Thus, MOPSO is verified to be efficient and practical in emergency communication scenarios, offering valuable insights for future deployments of aerial communication networks in disaster response situations.

## Figures and Tables

**Table 1:** Comparison with existing research.

Reference	Model	Objective Function	Methodology
[1]	Not specified.	Throughput, delay.	Not specified.
[4]	Not specified.	Quality of service (QoS).	Not specified.
[9]	Okumura-Hata model.	Coverage.	Not specified.
[10]	Rician channel model.	Maximization of sum rate.	Interference alignment technique.
[11]	Multi-layer placement model.	Minimize the number of balloons and maximize user connections.	Greedy algorithm and optimal algorithm.
[12]	Hata model.	Path loss, coverage area, and received signal strength.	Not specified.
[13]	Markov decision problem (MDP).	Maximization of total rate.	Resource allocation and placement control joint optimization scheme.
[14]	Ricean fading, free-space path loss (FSPL), and air-to-ground (ATG) channel model.	Maximize end-to-end throughput.	Joint layout and resource allocation algorithm.
[15]	Longley–Rice model.	Coverage.	Triangulation method.
[16]	Rayleigh fading, Nakagami-m fading, FSPL, and ATG model.	Maximize coverage probability.	Stochastic geometry tools and user association policy.
Our contribution	Gauss–Markov mobility model, FSPL, and ATG channel model.	Maximize target coverage and system energy efficiency.	MOPSO (Swarm intelligence optimizing multiple objectives).

**Table 2:** Primary simulation parameters.

Symbol	Definition	Value
$x_{min},x_{max},y_{min},y_{max}$	Simulation area	100 km × 100 km
$h_{min},h_{max}$	Minimum and maximum heights of balloons	400–1000 m
*N*	Number of balloons	1–10
*M*	Number of users	100
Λ	SINR threshold	8 dB
$f_{c}$	Carrier frequency	2.4 GHz
*B*	Total bandwidth	100 MHz
*c*	The speed of light	3 × 10^8^ m/s
$P_{min},P_{max}$	Minimum and maximum transmit power	60–100 W
$\eta_{LoS}$	Additional path loss under LoS	1 dB
$\eta_{NLoS}$	Additional path loss under NLoS	20 dB
*a*	Environmental impact factor	9.6
*b*	Environmental impact factor	0.2
$d_{min}$	Minimum distance between adjacent balloons	500 m

## Data Availability

The program code used in the research can be obtained from the corresponding author upon reasonable request.
